# A ‘borderline’ doctor: can you study medicine with a diagnosis of personality disorder?

**DOI:** 10.1192/bjb.2025.10173

**Published:** 2026-06

**Authors:** Erin McCabe, Paul A. Tiffin

**Affiliations:** 1 Diana Princess of Wales Hospital, Northern Lincolnshire and Goole NHS Foundation Trust, Grimsby, UK; 2 Hull York Medical School, University of York, York, UK; 3 Tees, Esk and Wear Valleys (TEWV) NHS Foundation Trust, Darlington, UK

**Keywords:** Personality disorders, education and training, psychotherapy, self-harm, trauma and stressor-related disorders

## Abstract

Little has been written regarding the experience of training in medicine with a diagnosis of a personality disorder. The stigma of personality disorders, evidenced even within psychiatry, potentially marginalises affected students and resident doctors. This article provides a first-hand account of the lead author’s (E.M.) lived experience of being a medical student with a diagnosis of emotionally unstable personality disorder (EUPD). Challenges that have been faced include a lack of understanding, limited literature about medical students and doctors with personality disorders, and derogatory attitudes. Despite this, the positive aspects of the diagnosis are recognised, through enhanced resilience and heightened emotional sensitivity, which can benefit patients.

Personality disorder, according to the ICD-11, describes people who chronically struggle with aspects of their emotional life and in relating to others.^1^ ICD-11, although no longer subcategorising personality disorders, retains a ‘borderline pattern specifier’. This relates to the diagnosis of ‘borderline personality disorder’, previously a subtype of ‘emotionally unstable personality disorder’ (EUPD) in ICD-10. This was characterised by impulsiveness (e.g. engaging in risky behaviours), unstable moods, intense but fragile relationships, poor sense of identity, self-harm and/or suicidality and persistent feelings of emptiness. It often, although not invariably, occurs in the context of a history of chronic childhood adversity or abuse. In personality disorder with the borderline pattern specifier, the world feels too intense, feelings are often overwhelming and life events can trigger extreme distress. The concept has its roots in the writings of the psychoanalyst Adolph Stern.^2^ Stern postulated that such individuals occupied a ‘borderline’ between neurosis (the tendency to experience negative feelings like anxiety and depression) and psychosis (characterised by a loss of contact with reality). The prognosis for EUPD is often described as poor, with an estimated 75% of individuals with this diagnosis attempting, and 10% completing, suicide.^3,4^

This article is written from the perspective of E.M., who was diagnosed with EUPD (subsequently, with ‘personality disorder with borderline specifier’) while a third-year medical student.

## The evidence base

Despite an estimated 4–15% of the population having a personality disorder,^5^ there is a dearth of literature about medical students and doctors with an EUPD diagnosis. I conducted a systematic search to identify literature involving a diagnosis of personality disorder in doctors, trainees and medical students. The search used the MEDLINE, Cochrane, PsycInfo and CINAHL databases from inception to March 2025. Terms relevant to personality disorder (personality disorder*, EUPD, BPD) and clinicians (physician*, clinician*, doctor*, medical student*) were combined. Of the 800 articles identified, some addressed clinician perceptions of EUPD, but no published research was identified that specifically addressed issues relating to a diagnosis of EUPD in clinicians or medical students.

Given this scarcity of relevant literature, I questioned whether I was the only medic with a diagnosis of personality disorder. Before my diagnosis, I heard such patients described as ‘manipulative’, ‘attention-seeking’ and heard derogatory comments such as ‘typical PD!’. Indeed, clinicians often view patients affected by personality disorders in a negative light and as less deserving of care.^6^ When exploring the attitudes of emergency medicine clinicians, comments revealed an uncomfortable personal reaction, with typical responses describing personality disorder as ‘an excuse [for] nastiness’ and people with the disorder as ‘manipulative’.^7^ Could the negative attitudes and perceptions regarding personality disorder explain the lack of literature on personality disorder in doctors? Is it the condition that ‘dare not speak its name’? I wondered if it was even possible to study medicine with the condition.

## Lived experience within medical education

I learned of my diagnosis on reading a letter to my general practitioner from a psychiatrist, it never having been discussed with me directly. This is common: over half of psychiatrists choose not to disclose such a diagnosis to their patients.^8^ I wondered why it had not been discussed with me, wondering if I too should avoid discussing it. I initially hid my diagnosis, worrying about the possible repercussions. Despite my struggles, I was always a diligent student, finding pride and fulfilment in my academic achievements, in exams, scholarship projects and essay competitions. Unfortunately, during my third year, my mental health deteriorated following a traumatic event. This culminated in a compulsory admission to hospital under the Mental Health Act. Medication is not a recommended treatment for the primary symptoms of personality disorder, although it can help coexisting mental health problems, in my case with some features of psychosis. Thankfully, I responded quickly to the medication I was started on, and I was discharged after only a couple of weeks.

Eager to return to my studies after the summer break, I didn’t fully process the scale of the deterioration in my symptoms, oscillating between a sense of being completely well or unwell. This struggle to accept a recovery period led to a student fitness to practise (FtP) referral. FtP is a terrifying concept for both medical students and doctors. Although frequently warned in medical school for missed deadlines and attendance or engagement issues, I never thought it would apply to me. My experience of an FtP investigation was difficult. At times it felt as if my future was being taken away, out of my reach. As with so many medical students, studying medicine formed a key part of my identity. Moreover, at the time the view of occupational health (OH) was that I was well enough to resume my studies. However, looking back, I was keen to appear well when seen by the OH doctor, so probably minimised my symptoms to some extent. Moreover, for these reasons, I declined the option to take a leave of absence to allow a period of recovery. This led to the Student FtP Committee declaring a ‘critical incident’ – effectively suspending my studies because of impaired fitness. Consequently, I undertook a year’s leave of absence, worried whether I could ever return to my studies. I was unsure how my condition was even viewed from an FtP perspective.

The threat of this loss and uncertainty led to initial worsening of my symptoms. However, with the support of an excellent community psychiatric nurse and my support network, I began to search for stability. I made a decision to put my full energy and focus into recovery, and began to fight for treatment. My access to therapy led to the start of my recovery, which gave rise to hope and I even started working as a mental health support worker. As I approached my FtP review I felt able to confront the concerns raised head on. The review was difficult; I felt vulnerable, understandably, given the stakes. Nevertheless, I was able to emotionally regulate and communicate effectively. I was declared ‘in Good Standing’ and allowed to resume my studies.

Box 1 gives my co-author P.A.T.’s reflections on my FtP referral.

Box 1 Reflections from a fitness to practise perspective (from co-author P.A.T., chair of the medical school Student Fitness to Practise Committee at the time)

A personality disorder diagnosis does not automatically imply impaired fitness to practise (FtP). Like all health conditions, the General Medical Council (GMC) expects medics to engage with appropriate treatment. This can be challenging, given limited availability of evidence-based treatments for personality disorder. Generally, such individuals pose most risk to themselves, via self-harm and risk-taking. However, the impact of emotion dysregulation on colleagues should be considered. Also, some patients may be distressed seeing evidence (such as scars) that a clinician or medical student has self-harmed.

Occupational health had reported that Erin was sufficiently recovered to return to her studies, and felt that refusing to let her do so might actually be detrimental to her well-being. However, the Student FtP Committee considered that her fitness to practise was impaired, given that substantial symptoms were still evident at an in-depth interview. However, Erin declined to take a leave of absence to allow time to recover. Consequently, the Committee suspended Erin’s studies, via the declaration of a ‘critical incident’, recommending appropriate treatment be accessed. At review, Erin’s improved insight and substantial progress resulted in a declaration of ‘Good Standing’. Recommendations for ongoing support were also made.

## Treatment for EUPD

Personality disorder is often seen as a ‘lifelong condition’. However, my access to life-changing treatment in the form of dialectical behaviour therapy (DBT) has helped enormously. DBT is an evidence-based treatment for borderline personality disorder, recommended by the National Institute for Health and Care Excellence (NICE).^9–11^ The therapy aims to develop skills in tolerating emotional distress, emotion regulation and interpersonal communication, helping the individual create a life worth living. A unique aspect of the DBT I received is the 24 h access to a ‘skills coach’, who helped implement my learning at difficult moments. I am now keen that others can also access this treatment, but there are barriers, including shortage of DBT providers. Also, in some services, access to this therapy requires a period free of self-injury – challenging for many patients to achieve.

There are further options for treatment, including schema-focused therapy, mentalisation-based therapy, interpersonal psychotherapy and transference-focused therapy. No approach has been proved to be superior to others.^12^ Although no single treatment appears to be the best choice in meta-analysis, DBT appeared to have the most solid evidence of effect.^13^ Despite the options for psychotherapy, almost 50% of people with a diagnosis of EUPD do not respond.^14^ It would be useful to explore direct head-to-head trials of therapies, to help establish an improved knowledge base for treatment choice.

## The negatives and the positives

Despite struggling with daily suicidal thoughts for some time, I knew I wanted to be alive and had a passion for medicine. From this struggle emerged empathy and a desire to help people at their lowest ebb. I did not know if this would be possible. Personality disorder still affects me, and likely always will. I am sensitive to even constructive criticism and must use my newfound skills to understand what is really meant by it. I still sometimes have urges to hurt myself, but can resist and do other, more positive things. I now recognise my own mental health issues and seek appropriate support at these times. In my medical practice, I will prioritise navigating challenging situations with a focus on balance. Medicine inherently presents circumstances that can evoke personal triggers, and this is something to which I am particularly attuned. In this, leveraging my positive coping strategies will be essential to maintaining both my well-being and my professional effectiveness.

There are, however, unexpected positive viewpoints that this disorder has brought. My heightened emotional sensitivity deepens my empathy, allowing me to connect with an individual’s feelings. Having experienced complex emotional states, I have increased my ability to problem-solve. It is not easy to manage some of my symptoms, but I am determined to do so. This fosters my resilience – identifying, solving and moving through intense states. I can see the positives in myself, but can also appreciate how this could make a difference for my patients. I am determined to make this experience a positive for my practice of medicine. I am grateful to have been recognised for my efforts and dedication, including being named Medical Student of the Year for Tees, Esk and Wear Valleys NHS Foundation Trust (a mental healthcare provider), receiving the award for best performance in clinical practice in my end-of-year exams, and being selected by the Royal College of Psychiatrists as a Psych Star for Liaison Psychiatry.

## Improving education and challenging stigma

I have also been ‘the patient’, and experienced a vast array of care, wide-ranging in quality. I know the fight patients can sometimes have to be listened to, and not just heard. There is stigma, both when accessing mental healthcare as a patient and when participating in a multidisciplinary team as a student. As with any patient group, we must have access to evidence-based treatments. For myself, and the many patients with a personality disorder, we need to be equal partners in our care. Indeed, the ‘invalidation’ and blame frequently experienced when help-seeking can exacerbate our difficulties and distress. Both undergraduate and postgraduate medical education should include more teaching on personality disorders and how such individuals may present and be supported. Teaching material should also enhance empathy and understanding of personality disorder. DBT frames personality disorder as valid, but ultimately unhealthy, entrenched learned ways of responding to early life adversity.

The lack of understanding and stigma surrounding personality disorders needs to be challenged. Throwaway comments from clinicians and educators can contribute to the ‘hidden curriculum’.^15^ This perpetuates the discrimination individuals affected by personality disorder face. For those supporting students with diagnosed or suspected personality disorder with the borderline pattern specifier, there are also interventions that are helpful. Difficulties in relationships is a common presenting symptom in personality disorders, therefore it could be useful for support to be consistent, with a single key person identified. Any changes to this must be managed sensitively, as a change of team members can be experienced as a re-enactment of loss or abandonment.^16^ I have found health passports to be helpful, which can appropriately share information with tutors on a need-to-know basis. Students should be signposted to appropriate services and reasonable adjustments made. For myself, I found meetings with a mentor to be useful. I also found use in adjustments for my exams, with rest breaks being helpful to allow time for regulation and distress tolerance skills to be used. With the right support I have been able to continue my studies, excelling at my exams.

I deliberated extensively on whether sharing my personal experiences was the right decision, especially having been previously cautioned about the potential implications for my future career. Ultimately, I chose to proceed. On receiving my diagnosis, the scarcity of accessible literature and first-hand accounts was unsettling, and I am committed to addressing this gap. Stigma perpetuates a damaging cyclical feedback loop: stigma leads to fear of disclosure, which in turn reinforces stigma. To break this cycle, I draw inspiration from individuals who have courageously shared their experiences of illness. For instance, Rebecca Lawrence’s openness about living with bipolar disorder has been both empowering and transformative, highlighting the value of personal narratives in reducing stigma and fostering understanding.^17^

Although my tolerance for stress is still lower than most ‘typical’ medical students, my experiences have fostered both empathy for others and personal resilience. These traits are invaluable to medicine. DBT draws its name from philosopher Hegel’s concept of a ‘dialectic’ – the process by which two seemingly opposing things can both be simultaneously true, with their synthesis giving rise to a new, third way: for example, the experience of embracing both ‘acceptance’ and the ‘need to change’. For me, this summarises my life journey, affected by EUPD while embracing a career in medicine.

## Data Availability

No new data were generated or analysed during this study.

## About the authors

**Erin McCabe** is a Foundation Year 1 Doctor at Diana Princess of Wales Hospital, Northern Lincolnshire and Goole NHS Foundation Trust, Grimsby, UK. **Paul A. Tiffin** is Professor of Psychiatry and Health Data Science at the Hull York Medical School, University of York, York, UK and an honorary consultant in the psychiatry of adolescence, Tees, Esk and Wear Valleys (TEWV) NHS Foundation Trust, Darlington, UK.
